# Pulmonary hypertension in the intensive care unit after pediatric allogeneic hematopoietic stem cell transplant: incidence, risk factors, and outcomes

**DOI:** 10.3389/fonc.2024.1415984

**Published:** 2024-05-29

**Authors:** Michael A. Smith, Geoffrey Cheng, Rachel Phelan, Ruta Brazauskas, Joelle Strom, Kwang Woo Ahn, Betty Ky Hamilton, Andrew Peterson, Bipin Savani, Hélène Schoemans, Michelle L. Schoettler, Mohamed Sorror, Roberta L. Keller, Christine S. Higham, Christopher C. Dvorak, Jeffrey R. Fineman, Matt S. Zinter

**Affiliations:** ^1^ Department of Pediatrics, Division of Critical Care Medicine, University of California, San Francisco, San Francisco, CA, United States; ^2^ Department of Pediatrics, Division of Pediatric Hematology/Oncology, University of California, San Francisco, San Francisco, CA, United States; ^3^ Center for International Blood and Marrow Transplant Research, Medical College of Wisconsin, Milwaukee, WI, United States; ^4^ Department of Hematology and Medical Oncology, Cleveland Clinic, Cleveland, OH, United States; ^5^ Department of Medicine, Vanderbilt University Medical Center, Nashville, TN, United States; ^6^ Department of Haematology, University Hospital Gasthuisberg, Leuven, Belgium; ^7^ Children’s Healthcare of Atlanta, Emory University, Atlanta, GA, United States; ^8^ Department of Hematology-Oncology, Fred Hutchinson Cancer Center, Seattle, WA, United States; ^9^ Department of Pediatrics, Division of Neonatology, University of California, San Francisco, San Francisco, CA, United States; ^10^ Department of Pediatrics, Division of Allergy, Immunology, and BMT, University of California, San Francisco, San Francisco, CA, United States

**Keywords:** pulmonary hypertension, stem cell transplant, pulmonary vascular disease, critical care, pediatrics

## Abstract

**Objective:**

To determine the incidence, risk factors, and outcomes of pulmonary hypertension (PH) in the pediatric intensive care unit (PICU) after pediatric hematopoietic stem cell transplant (HCT).

**Methods:**

This was a retrospective study of pediatric patients who underwent allogeneic HCT between January 2008-December 2014 at a center contributing to the Center for International Blood and Marrow Transplant Research data registry. Incidence of PH was assessed from PICU diagnostic codes from records merged from the Virtual Pediatric Systems database. Regression and survival analyses identified factors associated with post-HCT PH. Additional post-HCT morbidities and survival after PH were also assessed.

**Results:**

Among 6,995 HCT recipients, there were 29 cases of PH, a cumulative incidence of 0.42% (95% CI 0.27%-0.57%) at 60 months post-HCT. In the sub-cohort of 1,067 patients requiring intensive care after HCT, this accounted for a PH prevalence of 2.72% (95% CI 1.74–3.69%). There was an increased risk of developing PH associated with Black/African American race, metabolic disorders, partially HLA-matched or cord blood allografts, graft-versus-host prophylaxis regimen, and lower pre-HCT functional status. Patients who developed PH had significant PICU comorbidities including heart failure, pulmonary hemorrhage, respiratory failure, renal failure, and infections. Survival at 6 months after diagnosis of post-HCT PH was 51.7% (95% CI 32.5%-67.9%).

**Conclusions:**

PH is a rare but serious complication in the pediatric post-HCT population. A significant burden of additional comorbidities, procedural interventions, and risk of mortality is associated with its development. Close monitoring and prompt intervention for this severe complication are necessary in this vulnerable population.

## Introduction

Endothelial injury is a dominant pathologic process underlying a number of severe post-hematopoietic stem cell transplant (HCT) complications, including transplant-associated thrombotic microangiopathy (TA-TMA), hepatic veno-occlusive disease, idiopathic pneumonia syndrome, diffuse alveolar hemorrhage, and graft-versus-host disease (GVHD) (1). Endothelial dysfunction in the pulmonary vasculature in many disease states may manifest as pulmonary hypertension (PH), a pathology characterized by pulmonary vascular remodeling, elevated pulmonary arterial and right ventricular pressure, and eventually right heart failure leading to death. While cardiovascular diseases are reported to develop following pediatric HCT, pulmonary hypertension is one of the lesser studied despite contributing significantly to morbidity and mortality when arising in the post-transplant period (2, 3).

There are few studies that have examined post-HCT PH among children. These have focused primarily on high-risk populations and reported concerning outcomes. In 2013, Jodele, et al. reported a case series of 5 patients who were diagnosed with PH after developing hypoxemic respiratory failure post-HCT (4). Four of these patients died and all 3 who underwent autopsy demonstrated significant pulmonary vascular remodeling in the setting of TA-TMA. A 2019 study from Levy, et al. reported a retrospective review of 70 children who presented with unexplained respiratory symptoms after HCT, of which 22 (31%) were diagnosed with PH and 7/22 (32%) of these children suffered a fatal outcome (5). Finally, in 2023, a review of lung biopsy and postmortem samples from children with TA-TMA and respiratory failure identified 10 children with pulmonary vascular changes consistent with pulmonary arterial hypertension and/or pulmonary venous microthrombi, 9 (90%) of whom died (6). Each of these studies focused specifically on patients presenting with respiratory failure, but less is known about the incidence of PH and its outcomes among a broader representation of the transplant population.

Importantly, advanced medical therapies including novel therapeutic agents and critical care support technologies have improved outcomes in childhood PH (7). However, early identification and management of PH will be key in mitigating the poor outcomes that have been demonstrated among those who progress to respiratory failure prior to diagnosis of PH. Thus, a better understanding of the complication’s true epidemiology and identification of potential risk factors for PH among HCT recipients is needed. As such, we sought to address three aims. First, to report the cumulative incidence of PH during a PICU admission amongst a large, clinically diverse population of HCT recipients. Second, to describe the factors associated with its development in the post-HCT period. And lastly, to examine how PH affects long term survival following HCT.

## Methods

### Patient cohort

Two large administrative databases were merged to create the cohort analyzed in the present study. The Center for International Blood and Marrow Transplant Research (CIBMTR) comprises over 450 transplant centers worldwide and collects thorough data on consecutive allogeneic HCT patients. In addition to transplant-related data, high quality longitudinal follow up is included in the CIBMTR data collection. The Virtual Pediatric Systems (VPS) database documents consecutive PICU admissions to over 140 hospitals across North America. VPS records include patient demographics, International Classification of Diseases (ICD), Ninth and Tenth Revision and STAR diagnosis codes, severity of illness scores including the Pediatric Risk of Mortality-III (PRISM-III) score (8), and critical care interventions. STAR is the proprietary diagnosis classification schema of VPS. Trained VPS analysts assign diagnoses to patients based on review of attending physician documentation and ICD codes. Analysts collect admission information at each site with >95% inter-rater reliability.

Details of the CIBMTR and VPS database merge have been described previously (9, 10). In brief, CIBMTR records were collected for patients ≤21 years old who received a first allogeneic HCT between January 1, 2008 and December 31, 2014. Patients were excluded if they underwent HCT outside of the United States/Canada, had an identical twin donor, or lacked at least 100-day follow-up. Those who died within 100 days of HCT were included. VPS was then queried for patients ≤21 years of age admitted to a PICU between January 1, 2008 and December 31, 2014 with a diagnosis indicating prior HCT to derive a sub-cohort of post-transplant patients with critical illness. Short term semi-elective admissions (i.e., scheduled or perioperative admissions <2 days) were excluded. The HCT-related details from CIBMTR records were matched to patient records from the VPS database based on identical date of birth, sex, and transplant indication. This method was approved by the University of California, San Francisco (UCSF) institutional review board. An unblinded review of the matching results was performed from records at the UCSF Benioff Children’s Hospital and confirmed validity of matching (9).

### Outcomes

The primary outcome assessed was clinically significant PH, defined as PH requiring management in the PICU. Instances of the primary outcome were identified from STAR diagnosis codes. STAR codes included in the primary outcome were “416 Pulmonary Hypertension, Primary”, “416.8A Pulmonary Hypertension, Secondary”, and “416.9A Pulmonary Circulatory Disease” coded at any time during a PICU stay. Survival analyses examined all-cause mortality post-transplant.

### Predictors

Demographics, patient clinical characteristics, including HCT Comorbidity Index and Karnofsky/Lansky Performance Scores (11–13), and transplant-related factors were assessed as potential predictors of post-HCT PH.

### Statistical analysis

Descriptive statistics are reported as means with standard deviations and medians with interquartile ranges (IQR) as appropriate. Cumulative incidence of significant PH after HCT was determined using a cumulative incidence function, treating death as a competing event. Hazard ratios with 95% confidence intervals (CI) describing the risk of developing PH were derived for each predictor via univariate Cox proportional hazard models. Lastly, Kaplan-Meier curves were used to estimate the overall survival probabilities following HCT and following diagnosis of post-HCT PH.

## Results

### Baseline characteristics

There were 6,995 HCT recipients included in the final analyses. Patient demographics are reported in **Table 1**. Most patients underwent HCT for malignant diseases (57.4%), followed by non-malignant hematologic diseases (26.2%) and primary immunodeficiencies (11.5%). Most patients received bone marrow grafts (57.5%). Pre-transplant functional status was generally high, with most patients having a pre-transplant comorbidity index (11) of 0 (66.3%) and Karnofsky/Lansky performance score (12, 13) of 100 (53.1%). Median follow-up of survivors was 73 months (IQR 60–96 months).

**Table 1:** Baseline patient and transplant characteristics.

		Total, 6995	No PH, 6966 (99.6)	PH, 29 (0.4)
**Age (mean (SD))**		9.14 (6.17)	9.15 (6.17)	6.90 (6.03)
**Age group**	<1 year	665 (9.5)	659 (9.5)	6 (20.7)
	1–4 years	1598 (22.8)	1590 (22.8)	8 (27.6)
	5–12 years	2505 (35.8)	2496 (35.8)	9 (31.0)
	13–20 years	2227 (31.8)	2221 (31.9)	6 (20.7)
**Sex, female**		2893 (41.3)	2882 (41.3)	11 (37.9)
**Race**	White	5008 (75.8)	4989 (75.8)	19 (67.9)
	American Indian or Alaska Native	62 (0.9)	62 (0.9)	0 (0.0)
	Asian	356 (5.4)	356 (5.4)	0 (0.0)
	Black or African American	987 (14.9)	978 (14.9)	9 (32.1)
	Native Hawaiian or other Pacific Islander	17 (0.3)	17 (0.3)	0 (0.0)
	More than one race	179 (2.7)	179 (2.7)	0 (0.0)
	Missing	386	385	1
**Ethnicity, Hispanic or Latino**		1646 (24.3)	1640 (24.4)	6 (20.7)
Missing		232	232	0
**Insurance**	Private/military/dual insurance	1801 (59.6)	1797 (59.6)	4 (36.4)
	Public insurance only	1169 (38.7)	1162 (38.6)	7 (63.6)
	Uninsured	54 (1.8)	54 (1.8)	0 (0.0)
	Missing	3971	3953	18
**Neighborhood median household income (median [IQR])**		$52,348 [41,323, 68,774]	$52,348 [41,332, 68,795]	$53,298 [38,198, 62,209]
**BMI (median [IQR])**		18.34 [16.23, 22.06]	18.34 [16.23, 22.06]	18.90 [17.56, 24.31]
**BMI Classification**	Normal	2159 (58.7)	2153 (58.8)	6 (46.2)
	Overweight	601 (16.4)	598 (16.3)	3 (23.1)
	Obese	643 (17.5)	639 (17.4)	4 (30.8)
	Underweight	272 (7.4)	272 (7.4)	0 (0.0)
	Missing	3320	3304	16
**Indication for transplant**	Malignant disease	4013 (57.4)	3998 (57.4)	15 (51.7)
	Non-malignant hematologic disease	1833 (26.2)	1827 (26.2)	6 (20.7)
	Metabolic disorders	324 (4.6)	320 (4.6)	4 (13.8)
	Primary immunodeficiency	805 (11.5)	801 (11.5)	4 (13.8)
	Other disease	20 (0.3)	20 (0.3)	0 (0.0)
**HCT comorbidity index**	0	4638 (66.8)	4623 (66.8)	15 (51.7)
	1	910 (13.1)	910 (13.2)	0 (0.0)
	2	384 (5.5)	382 (5.5)	2 (6.9)
	3+	1016 (14.6)	1004 (14.5)	12 (41.4)
	Missing	47	47	0
**Karnofsky score**	100	3714 (54.1)	3700 (54.2)	14 (48.3)
	90	2164 (31.5)	2159 (31.6)	5 (17.2)
	<=80	982 (14.3)	972 (14.2)	10 (34.5)
	Missing	135	135	0
**Conditioning regimen**	RIC/NMA	1793 (25.8)	1788 (25.8)	5 (17.2)
	MAC-No TBI	2675 (38.4)	2662 (38.4)	13 (44.8)
	MAC-TBI	2436 (35.0)	2425 (35.0)	11 (37.9)
	No conditioning	57 (0.8)	57 (0.8)	0 (0.0)
	Missing	34	34	0
**ATG/Alemtuzumab conditioning**	Neither	2770 (50.5)	2760 (50.5)	10 (45.5)
	ATG alone	2706 (49.3)	2694 (49.3)	12 (54.5)
	Alemtuzumab alone	12 (0.2)	12 (0.2)	0 (0.0)
	Missing	1507	1500	7
**Graft type**	Bone marrow	4022 (57.5)	4009 (57.6)	13 (44.8)
	Cord blood	1911 (27.3)	1898 (27.2)	13 (44.8)
	Peripheral blood	1062 (15.2)	1059 (15.2)	3 (10.3)
**HLA matching**	HLA-identical sibling	1860 (26.6)	1857 (26.7)	3 (10.3)
	Well-matched unrelated (8/8)	1888 (27.0)	1885 (27.1)	3 (10.3)
	Partially matched related	429 (6.1)	426 (6.1)	3 (10.3)
	Partially matched unrelated	830 (11.9)	823 (11.8)	7 (24.1)
	Cord blood	1911 (27.3)	1898 (27.3)	13 (44.8)
	Missing	77	77	0
**Sex matching**	Matched	2762 (39.5)	2756 (39.6)	6 (20.7)
	Mismatch	2311 (33.1)	2301 (33.1)	10 (34.5)
	Cord blood	1911 (27.4)	1898 (27.3)	13 (44.8)
	Missing	11	11	0
**Recipient CMV status, positive**		3872 (56.3)	3852 (56.2)	20 (71.4)
Missing		116	115	1
**GVH prophylaxis regimen**	CNI + MTX	3316 (47.4)	3308 (47.5)	8 (27.6)
	CNI + MMF	2117 (30.3)	2103 (30.2)	14 (48.3)
	CNI +/- others	1049 (15.0)	1044 (15.0)	5 (17.2)
	TCD	349 (5.0)	347 (5.0)	2 (6.9)
	Other/missing	164 (2.4)	164 (2.4)	0 (0.0)
**Acute GVHD (max grade)**	None	3629 (51.9)	3609 (51.8)	20 (69.0)
	Grade I	926 (13.2)	923 (13.3)	3 (10.3)
	Grade II	1170 (16.7)	1167 (16.8)	3 (10.3)
	Grade III	673 (9.6)	671 (9.6)	2 (6.9)
	Grade IV	373 (5.3)	373 (5.4)	0 (0.0)
	Present, grade unknown	214 (3.1)	214 (3.1)	0 (0.0)
	Missing	10	9	1
**Chronic GVHD (max grade)**	None	5035 (72.0)	5010 (71.9)	25 (86.2)
	Limited	759 (10.9)	757 (10.9)	2 (6.9)
	Extensive	1180 (16.9)	1178 (16.9)	2 (6.9)
	Present, grade unknown	2 (0.0)	0 (0.0)	0 (0.0)
	Missing	19	19	0

Values represent n (%) unless otherwise indicated. RIC (Reduced Intensity Chemotherapy). NMA (Non-Myeloablative). TBI (Total Body Irradiation). ATG (Anti-Thymocyte Globulin). HLA (Human Leukocyte Antigen). CNI (Calcineurin Inhibitor). MTX (Methotrexate). MMF (Mycophenolate Mofetil). TCD (T Cell Depletion). GVHD (Graft Versus Host Disease).

### Cumulative incidence and risk factors for PH

**Figure 1:**
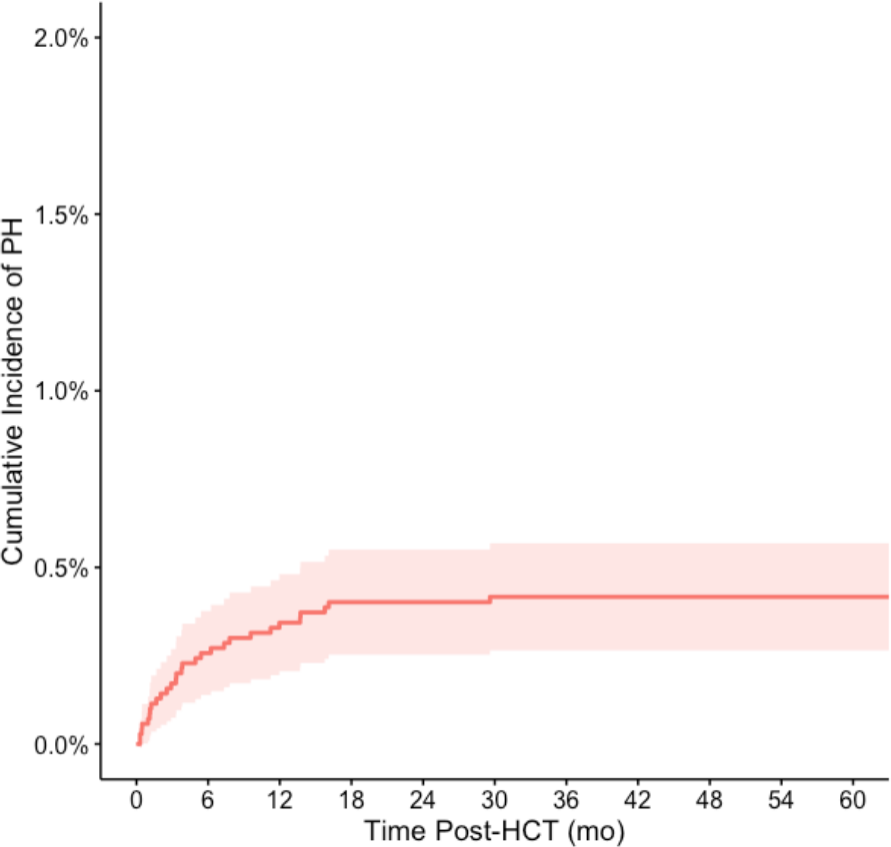
Cumulative incidence of pulmonary hypertension in the PICU following stem cell transplant. The cumulative incidence of pulmonary hypertension following HCT was 0.42% (95% CI 0.27%-0.57%) at 60 months post-HCT. Death was treated as a competing event in the cumulative incidence calculation.

**Figure 2:**
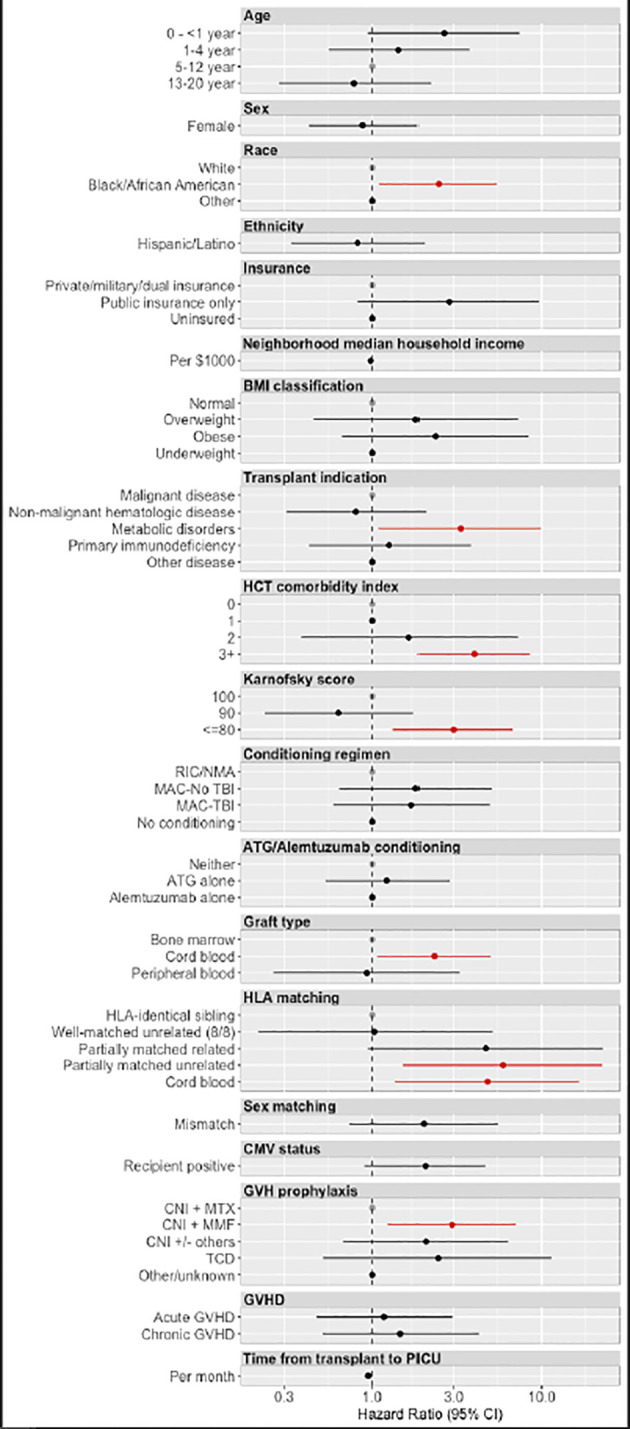
Hazard ratios for the development of pulmonary hypertension. Hazard ratios for the development of pulmonary hypertension were derived from univariable Cox regression models. Factors associated with statistically significant increased risk for post-transplant pulmonary hypertension included Black/African American race, metabolic disorders as the primary transplant indication, worse HCT comorbidity index and Karnofsky scores, partial HLA matching in unrelated donors, cord blood transplants, and CNI + MMF GVH prophylaxis regimens. RIC (Reduced Intensity Chemotherapy). NMA (Non-Myeloablative). TBI (Total Body Irradiation). ATG (Anti-Thymocyte Globulin). HLA (Human Leukocyte Antigen). CNI (Calcineurin Inhibitor). MTX (Methotrexate). MMF (Mycophenolate Mofetil). TCD (T Cell Depletion). GVHD (Graft Versus Host Disease).

There were 29 total cases of the primary outcome of PH managed in the PICU. Among all HCT recipients, 5-year cumulative incidence of significant PH was 0.42% (95% CI 0.27%-0.57%; **Figure 1**). Variables associated with the development of post-HCT PH are depicted in **Figure 2** (**Supplementary Table 1**). There was an increased risk of developing significant PH in Black/African American patients relative to White (HR 2.44, 95% CI 1.10–5.40 p=0.027), patients being transplanted for metabolic disorders relative to malignancies (HR 3.30, 95% CI 1.09–9.93, p=0.034), those who received partially HLA-matched unrelated or cord blood grafts relative to grafts from HLA-identical siblings (HR 5.89, 95% CI 1.52–22.79, p=0.010; HR 4.76, 95% CI 1.36–16.72, p=0.015, respectively), as well as those who received cord blood grafts relative to bone marrow (HR 2.30, 95% CI 1.07–4.97, p=0.033). Those with worse pre-transplant functional status had higher risk of significant PH. Patients with a comorbidity index of 3+ demonstrated 3.98 times the risk (95% CI 1.86–8.51, p<0.001) compared to those with a comorbidity index of 0, and those with a Karnofsky/Lansky score of ≤80 demonstrated 2.99 times the risk (95% CI 1.33–6.74, p=0.008) compared to a score of 100. Patients who received GVHD prophylaxis with a calcineurin inhibitor plus mycophenolate mofetil (MMF) had an increased risk of significant PH compared to those treated with a calcineurin inhibitor and methotrexate (HR 2.93, 95% CI 1.23–6.98, p=0.015). There were no statistically significant differences in the risk of developing significant PH based on age, sex, insurance status, BMI classification, conditioning regimens, sex matching, or post-HCT GVHD status.

### PICU characteristics of PH patients

There were 1,067 patients admitted to the PICU post-transplant for a total of 2,107 admissions. The 29 patients diagnosed with significant PH were admitted to the PICU for a total of 37 admissions. Among the sub-cohort of post-HCT patients with critical illness, the prevalence of PH was 2.72% (95% CI 1.74–3.69%). The median time from HCT to PICU admission with PH was 3.77 months (IQR l.23–9.57 months) which was comparable to the median time between HCT and PICU admission for non-PH critical illness (p=0.17). Additional PICU-related characteristics of the PH patients at the time of PH diagnosis are described in **Supplementary Table 2**. The majority of patients were admitted with a PRISM-3 score >10 (n=14, 51.9%), although some patients had low PRISM-3 scores at the time of PICU admission (e.g. score 0–2 in n=7, 25.9%). Comorbidities observed during the PICU stays included heart failure (n=5, 17.2%), pulmonary hemorrhage (n=7, 24.1%), thrombotic microangiopathy (n=1, 3.4%), hepatobiliary failure (n=3, 10.3%), and renal failure (n=11, 37.9%). Infections were relatively common (bacterial n=15, 51.7%; viral n=14, 48.3%; and fungal n=5, 17.2%). Intubation was required in 21 patients (72.4%) and renal replacement therapy occurred in 8 (27.6%).

### Post-transplant mortality

**Figure 3:**
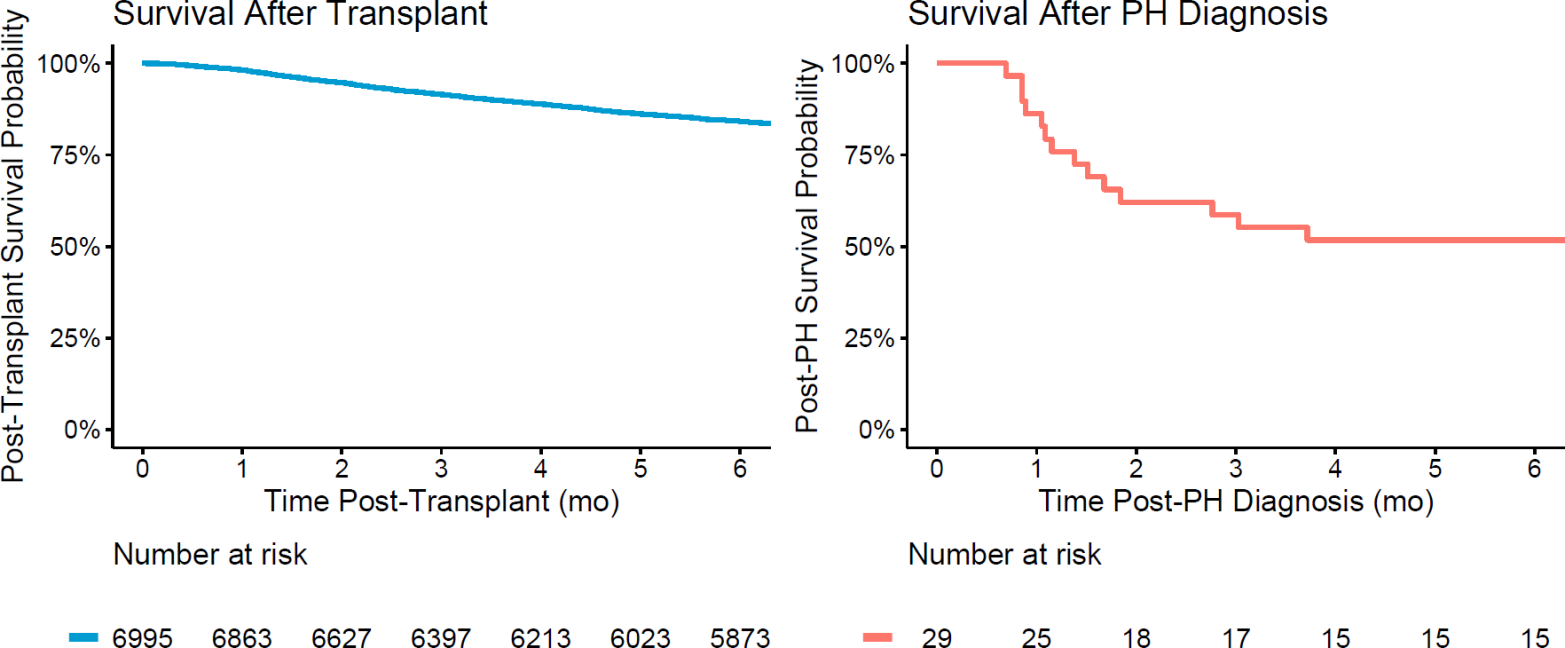
Survival following HCT and following diagnosis of post-HCT PH. Overall survival at 6 months following HCT was 84.1% (95% CI 83.2%-85.0%). Overall survival at 6 months following diagnosis of PH was 51.7% (95% CI 32.5%-67.9%).

During the study period, 16 of the 29 patients with PH died. Most deaths occurred early, within 3 months after PH onset, and all occurred during a PICU admission, with 15 of the 16 occurring in the patient’s first post-HCT PICU admission. The overall survival at 6 months following diagnosis of PH was 51.7% (95% CI 32.5%-67.9%, **Figure 3**). Death was attributed to primary disease in 5 cases, organ failure or infection in 4 cases each, and GVHD, hemorrhage, or other causes in 1 case each. Among the entire cohort, overall survival at 6 months post-HCT was 84.1% (95% CI 83.2%-85.0%).

## Discussion

Our findings provide new insight into the epidemiology, risk factors, and prognosis of significant PH following pediatric HCT. The incidence of significant post-HCT PH is low among the entire allogeneic HCT population, estimated at 0.42% (95% CI 0.27%-0.57%) at 60 months post-HCT. Among the patients requiring PICU care post-HCT, PH was prevalent in 2.72%. Those who developed PH had notable intensive care comorbidities and required significant invasive interventions. Post-PH mortality was significant and occurred early after diagnosis. These findings provide an updated understanding of a rare post-HCT complication and could serve as a benchmark for future studies.

Prior reports examining the overall incidence of post-HCT PH have cited rates as high as 15–28% among children (5, 14, 15). However, these studies focused only on subsets of the HCT population who were transplanted for specific diseases, such as osteopetrosis or CNS tumors. Studies including a wider breadth of transplant recipients have primarily examined PH incidence among those who have already developed cardiorespiratory symptoms and thus represent an enriched population (4, 5, 16). As such, by studying all allogeneic transplant recipients from the time of transplant, our study found a significantly lower cumulative incidence of post-HCT PH.

It is difficult to define a comprehensive pre-transplant risk profile for PH with such a low incidence of disease. Nonetheless, we were able to identify several factors associated with its development. African American patients, patients with a poor pre-HCT Comorbidity Index or Karnofsky/Lansky score, and patients with metabolic disorders had increased risk for PH. Racial differences in pediatric PH have been reported previously, including one study finding that Black children demonstrate an increased odds of lung disease-associated PH (17). PH has also been previously associated with inborn errors of metabolism, primarily those related to mitochondrial dysfunction, cobalamin C defects, and mucopolysaccharidoses (18, 19). Patients with mucopolysaccharidoses carry an increased baseline risk of developing pulmonary vascular disease due to the pathologic deposition of glycosaminoglycans leading to obstructive airway, restrictive lung, and valvular heart diseases (20). The exact reasons underlying the increased risk of PH for these groups in our cohort cannot be elucidated due to the limitations of our data set. Nonetheless, these factors may help guide future investigations.

The contribution of alloreactivity to post-HCT PH remains uncertain. Although neither acute nor chronic GVHD were associated with PH, PH was associated with both the use of partially matched unrelated donors and the use of calcineurin inhibitor and mycophenolate mofetil (CNI + MMF) for GVHD prophylaxis (both of which are associated with greater rates of GVHD (21)). Similar results have been reported previously (6). This alludes to the possibility that GVHD-mediated injury may underlie the endothelial dysfunction that contributes to PH in these rare patients. Prior studies have reported that certain conditioning agents may be associated with increased risk of endothelial dysfunction and PH (1, 5). Unfortunately, we were unable to examine the associations between specific agents and PH in this data set. Further studies examining the mechanisms of endothelial dysfunction and pulmonary vascular disease after HCT are needed to better understand who will be at greatest risk for developing these complications.

Patients with PH experienced notable comorbidities during their PICU stays, including high rates of infection and multiple different organ system failures that often required invasive interventions. Interestingly, PRISM-3 risk of mortality scores for critically ill patients did not universally reflect the severity of the clinical course for all patients. Nine of the 29 patients were admitted with PRISM-3 scores less than 5. The PRISM-3 score is an established tool that has demonstrated excellent predictive power, with an area under receiver operating curve of 0.95 for predicting PICU mortality (8). In the pediatric HCT population, it is included as one of five components of a focused mortality risk prediction model that demonstrated improved performance in this unique sub-population (9). Despite this, it failed to consistently predict the significantly worse prognosis of those with PH in our cohort. Failure to identify high risk patients early on is one of the major limitations in a number of other studies examining this vulnerable population (9, 22–24). Our findings enforce the need for development of alternative prognostication models that incorporate early features of disease, potentially including echocardiographic or blood biomarkers, and transplant-related factors to better identify early organ dysfunction and risk of mortality among this unique subset of the PICU population. This is particularly important in the setting of post-HCT PH, where early PH identification and management has been demonstrated to significantly improve outcomes for these children (5).

Our study has a number of strengths. A large, diverse population of allogeneic transplant recipients from around North America was included for analysis and the merging of VPS PICU data with CIBMTR transplant data provides a valuable and unique level of detail to the analyses. There were also several limitations to our study. First, we were limited in our assessment of post-transplant PH to cases that required PICU admission and specifically documented a PH diagnosis. Detailed echocardiographic or catheterization data were not available nor was there a uniform screening protocol to identify all cases. Therefore, this report likely underestimates the true incidence of post-HCT PH, and probably fails to take into account mild cases. Lastly, the low incidence of disease limited our abilities to form a comprehensive predictive model due to lack of statistical power.

In summary, we have found that the incidence of clinically significant PH developing after pediatric allogeneic HCT is likely low, though prospective studies employing a standardized screening protocol are needed to confirm the true incidence rate. For those who develop PH and require intensive care, there is a significant burden of PICU morbidity and post-transplant mortality. Future studies should continue to focus efforts on understanding the clinical course and underlying pathophysiology of the rare but serious post-HCT complication of PH.

## Data availability statement

The original contributions presented in the study are included in the article/**Supplementary Material**. Further inquiries can be directed to the corresponding author and CIBMTR at info-request@mcw.edu.

## Author contributions

MSm: Writing – original draft, Conceptualization, Formal analysis, Methodology. GC: Conceptualization, Formal analysis, Methodology, Writing – review & editing. RP: Writing – review & editing. RB: Writing – review & editing. JS: Writing – review & editing. KA: Writing – review & editing. BH: Writing – review & editing. AP: Writing – review & editing. BS: Writing – review & editing. HS: Writing – review & editing. MSc: Writing – review & editing. MSo: Writing – review & editing. RK: Writing – review & editing. CH: Writing – review & editing. CD: Writing – review & editing. JF: Writing – review & editing. MZ: Writing – original draft.
