# Supplementary material for: Pulmonary hypertension in the intensive care unit after pediatric allogeneic hematopoietic stem cell transplant: incidence, risk factors, and outcomes
Source: Front Oncol. 2024 May 29;14:1415984. doi: 10.3389/fonc.2024.1415984 (PMC11167102; doi:10.3389/fonc.2024.1415984)
Supplement: Supplementary file 1 [file Table_1.docx]

Supplementary Material

Pulmonary Hypertension After Pediatric Allogeneic Hematopoietic Stem Cell Transplant: Incidence, Risk Factors, and Outcomes

Michael A. Smith*, Geoffrey Cheng, Rachel Phelan, Ruta Brazauskas, Joelle Strom, Kwang Woo Ahn, Betty Hamilton, Andrew Peterson, Bipin Savani, Hélène Schoemans, Michelle Schoettler, Mohamed Sorror, Roberta L. Keller, Christine S. Higham, Christopher C. Dvorak, Jeffrey R. Fineman, Matt S. Zinter

*** Correspondence:** Michael Smith: michael.smith2@ucsf.edu

**Supplemental Table 1**

|  | | | |  | | | | | | | | | | **Hazard Ratio**  **(95% Confidence Interval)** | | **p value** |
| --- | --- | --- | --- | --- | --- | --- | --- | --- | --- | --- | --- | --- | --- | --- | --- | --- |
|  | | | |  | | | | | | | | | |  |  |  |
| **Age group** | | | | <1 year | | | | | | | | | | 2.63 (0.94-7.39) | | 0.07 |
|  | | | | 1-4 years | | | | | | | | | | 1.43 (0.55-3.71) | | 0.46 |
|  | | | | 5-12 years | | | | | | | | | | Reference |  | |
|  | | | | 13-20 years | | | | | | | | | | 0.78 (0.28-2.20) | | 0.64 |
| **Sex, female** | | | |  | | | | | | | | | | 0.88 (0.42-1.86) | | 0.74 |
| **Race** | | | | White | | | | | | | | | | Reference |  | |
|  | | | | | Black or African American | | | | | | | | | 2.44 (1.10-5.40) | | 0.027* |
|  | Other | | | | | | | | | | | | | NA | | NA |
| **Ethnicity, Hispanic or Latino** | | | | | | | | | | | | | | 0.82 (0.33-2.02) | | 0.67 |
| **Insurance** | | | Private/military/dual insurance | | | | | | | | | | | Reference |  | |
|  | | | | Public insurance only | | | | | | | | | | 2.82 (0.82-9.63) | | 0.10 |
|  | | | | Uninsured | | | | | | | | | | NA | | NA |
| **Neighborhood median household income (per $1000)** | | | | | | | | | | | | | | 0.98 (0.96-1.01) | | 0.11 |
| **BMI Classification** | | | | | | Normal | | | | | | | | Reference |  | |
|  | | | | Overweight | | | | | | | | | | 1.81 (0.45-7.25) | | 0.40 |
|  | | | | Obese | | | | | | | | | | 2.34 (0.66-8.30) | | 0.19 |
|  | | | | Underweight | | | | | | | | | | NA | | NA |
| **Indication for transplant** | | | | | | | | | | Malignant disease | | | | Reference |  | |
|  | | Non-malignant hematologic disease | | | | | | | | | | | | 0.80 (0.31-2.07) | | 0.65 |
|  | | | | Metabolic disorders | | | | | | | | | | 3.30 (1.09-9.93) | | 0.034* |
|  | | | | | Primary immunodeficiency | | | | | | | | | 1.26 (0.42-3.79) | | 0.68 |
|  | | | | Other disease | | | | | | | | | | NA | | NA |
| **HCT comorbidity index** | | | | | | | | | | | 0 | | | Reference |  | |
|  | | | | 1 | | | | | | | | | | NA | | NA |
|  | | | | 2 | | | | | | | | | | 1.65 (0.38-7.23) | | 0.50 |
|  | | | | 3+ | | | | | | | | | | 3.98 (1.86-8.51) | | <0.001* |
| **Karnofsky score** | | | | | | | 100 | | | | | | | Reference |  | |
|  | | | | 90 | | | | | | | | | | 0.63 (0.23-1.75) | | 0.37 |
|  | | | | <=80 | | | | | | | | | | 2.99 (1.33-6.74) | | 0.008* |
| **Conditioning regimen** | | | | | | | | RIC/NMA | | | | | | Reference |  | |
|  | | | | MAC-No TBI | | | | | | | | | | 1.81 (0.64-5.06) | | 0.26 |
|  | | | | MAC-TBI | | | | | | | | | | 1.70 (0.59-4.90) | | 0.32 |
|  | | | | No conditioning | | | | | | | | | | NA | | NA |
| **ATG/Alemtuzumab conditioning** | | | | | | | | | | | | Neither | | Reference |  | |
|  | | | | ATG alone | | | | | | | | | | 1.22 (0.53-2.83) | | 0.64 |
|  | | | | Alemtuzumab alone | | | | | | | | | | NA | | NA |
| **Graft type** | | | | Bone marrow | | | | | | | | | | Reference |  | |
|  | | | | Cord blood | | | | | | | | | | 2.30 (1.07-4.97) | | 0.033* |
|  | | | | Peripheral blood | | | | | | | | | | 0.93 (0.26-3.26) | | 0.91 |
| **HLA matching** | | | | | | | | | HLA-identical sibling | | | | | Reference |  | |
|  | | | | Well-matched unrelated (8/8) | | | | | | | | | | 1.03 (0.21-5.12) | | 0.97 |
|  | | | | Partially matched related | | | | | | | | | | 4.65 (0.94-23.04) | | 0.06 |
|  | | | | | | Partially matched unrelated | | | | | | | | 5.89 (1.52-22.79) | | 0.010* |
|  | | | | Cord blood | | | | | | | | | | 4.76 (1.36-16.72) | | 0.015* |
| **Sex matching** | | | | | | | | | | Mismatch | | | | 2.00 (0.73-5.50) | | 0.18 |
| **Recipient CMV status, positive** | | | | | | | | | | | | | | 2.04 (0.90-4.63) | | 0.09 |
| **GVH prophylaxis regimen** | | | | | | | | | | CNI + MTX | | | | Reference |  | |
|  | | | | CNI + MMF | | | | | | | | | | 2.93 (1.23-6.98) | | 0.015* |
|  | | | | CNI +/- others | | | | | | | | | | 2.05 (0.67-6.28) | | 0.21 |
|  | | | | TCD | | | | | | | | | | 2.42 (0.51-11.41) | | 0.26 |
|  | | | | Other/unknown | | | | | | | | | | NA | | NA |
| **GVHD** | | | | Acute | | | | | | | | | | 1.17 (0.47-2.94) | | 0.73 |
|  | | | | Chronic | | | | | | | | | | 1.47 (0.51-4.24) | | 0.48 |
| **Time from transplant to PICU (per month)** | | | | | | | | | | | | |  | 0.95 (0.90-1.01) | | 0.11 |

Hazard ratios for the development of pulmonary hypertension were derived from univariate Cox regression models. GVHD was treated as a time-varying covariate. Factors associated with statistically significant increased risk for post-transplant pulmonary hypertension included Black/African American race, metabolic disorders as the primary transplant indication, worse HCT comorbidity index and Karnofsky scores, partial HLA matching in unrelated donors, cord blood transplants, and CNI + MMF GVH prophylaxis regimens. * indicates significant p values <0.05. NA indicates no PH occurred in the specified category.

**Supplemental Table 2**

|  | | | |  | | | **PH Patients, 29** |
| --- | --- | --- | --- | --- | --- | --- | --- |
| **Age at PICU admission** | | | | | | <1 year | 5 (17.2) |
|  | | | | | | 1-4 years | 9 (31.0) |
|  | | | | | | 5-12 years | 8 (27.6) |
|  | | | | 13-20 years | | | 7 (24.1) |
| **Time from transplant to first PICU admission (months), median (IQR)** | | | | | | | 3.77 (1.23, 9.57) |
| **PRISM-3 Score** | | | | 0-2 | | | 7 (25.9) |
|  | | | | 3-5 | | | 2 (7.4) |
|  | | | | 6-10 | | | 4 (14.8) |
|  | | | | >10 | | | 14 (51.9) |
| **Comorbidities** | | | | Hypertension | | | 10 (34.5) |
|  | | | | Cardiomyopathy | | | 1 (3.4) |
|  | | | | Pericardial disease | | | 3 (10.3) |
|  | | | | Heart failure | | | 5 (17.2) |
|  | | | | Pulmonary hemorrhage | | | 7 (24.1) |
|  | | | | Gastrointestinal mucositis | | | 1 (3.4) |
|  | Failure to thrive/malnutrition | | | | | | 3 (10.3) |
|  | | | Thrombotic microangiopathy | | | | 1 (3.4) |
|  | | | | Renal failure | | | 11 (37.9) |
|  | | | | Hepatobiliary failure | | | 3 (10.3) |
|  | | | | Bacterial infection | | | 15 (51.7) |
|  | | | | Viral infection | | | 14 (48.3) |
|  | | | | Fungal infection | | | 5 (17.2) |
|  | | | | | Infection, unspecified | | 17 (58.6) |
| **Procedures** | | | | Intubation | | | 21 (72.4) |
|  | | | | Renal replacement therapy | | | 8 (27.6) |
|  | | Extracorporeal membrane oxygenation | | | | | 1 (3.4) |

Clinical characteristics, comorbidities, and interventions during the first PICU admission of patients with PH. Values represent n (%) unless otherwise indicated.
